# Non-schizophrenic psychotic disorders: Cycloid psychosis. Case report and literature review

**DOI:** 10.1192/j.eurpsy.2023.1323

**Published:** 2023-07-19

**Authors:** P. Herrero Ortega, J. Garde González, M. A. Morillas Romerosa, A. Oliva Lozano, J. Curto Ramos

**Affiliations:** Psychiatry, La Paz University Hospital, Madrid, Spain

## Abstract

**Introduction:**

Cycloid psychosis is a clinical entity with defining traits which emerged from the Wernicke-Kleist-Leonhard school of psychiatry and has a long history in Europe. Leonhard distinguished three clinical forms: anxiety-happiness, confusion and motility psychosis. It is a condition with high clinical heterogeneity and favorable prognosis.

**Objectives:**

To describe a case of cycloid psychosis and review in literature the heterogeneity of this phenomena and its clinical management.

**Methods:**

Clinical case report and brief review of literature.

**Results:**

57-year old male with previous diagnosis of paranoid schizophrenia and severe congenital hearing loss. Preserved autonomy and adequate real-life and interpersonal functioning. Along the past few years the patient has presented episodes of a significant clinical global worsening in context of mainly somatic symptoms (intestinal obstruction and volvulus) and minor stressful life events. On this occasion he appears in the emergency room with a new episode of abdominal pain and is admitted to Internal Medicine with presumptive diagnosis of intestinal volvulus. The patient gathered heterogeneous symptoms including disorientation, confusion, generalized tremor, gait disorder, profuse sweating, regressive and oppositional behaviors (refusal to eat or drink liquids) and sudden behavioral oscillations (from agitation to prostration). From the psychic point of view he showed thought blocking, mutism, significant anxiety, fear of death, delusional prejudice ideas and sensoperceptive disturbance which seemed otherwise related to previous sensorial problems. We introduced treatment with Olanzapine 30 mg and after 4 weeks, the patient suddenly showed a significant clinical improvement until the complete remission of the symptoms and restitution of his previous state. In coordination with his regular psychiatrist was proposed the controversial diagnosis of cycloid psychosis. Cycloid psychosis gather a few clinical features which differentiate it from other entities: acute onset, polymorphic symptomatology, global disturbance of psychic life, remitting and recurrent course and favorable prognosis. Regarding its clinical management no controlled studies have been conducted to this date of the treatment of this phenomena. According to literature ECT seems to be an effective treatment as well as low-doses of atypical antipsychotics. Some authors propose pharmacological maintenance treatment with mood stabilizers.

**Conclusions:**

The diagnosis of cycloid psychosis can be useful as well as necessary to describe certain patients with similar clinical features, recurrent course and favorable prognosis. Future studies on pharmacological approach would be useful to ensure the appropriate clinical management of these patients.

**Disclosure of Interest:**

None Declared

